# The Great Debate: General Ability and Specific Abilities in the Prediction of Important Outcomes

**DOI:** 10.3390/jintelligence6030039

**Published:** 2018-09-07

**Authors:** Harrison J. Kell, Jonas W. B. Lang

**Affiliations:** 1 Academic to Career Research Center, Research & Development, Educational Testing Service, Princeton, NJ 08541, USA; 2 Department of Personnel Management, Work, and Organizational Psychology, Ghent University, Henri Dunantlaan 2, 9000 Ghent, Belgium

**Keywords:** bifactor model, cognitive abilities, educational attainment, general mental ability, hierarchical factor model, higher-order factor model, intelligence, job performance, nested-factors model, relative importance analysis, specific abilities

## Abstract

The relative value of specific versus general cognitive abilities for the prediction of practical outcomes has been debated since the inception of modern intelligence theorizing and testing. This editorial introduces a special issue dedicated to exploring this ongoing “great debate”. It provides an overview of the debate, explains the motivation for the special issue and two types of submissions solicited, and briefly illustrates how differing conceptualizations of cognitive abilities demand different analytic strategies for predicting criteria, and that these different strategies can yield conflicting findings about the real-world importance of general versus specific abilities.

## 1. Introduction to the Special Issue

“To state one argument is not necessarily to be deaf to all others.”

—Robert Louis Stevenson [1] (p. 11).

Measuring intelligence with the express purpose of predicting practical outcomes has played a major role in the discipline since its exception [2]. The apparent failure of sensory tests of intelligence to predict school grades led to their demise [3,4]. The Binet-Simon [5] was created with the practical goal of identifying students with developmental delays in order to track them into different schools as universal public education was instituted in France [6]. The Binet-Simon is considered the first “modern” intelligence test because it succeeded in fulfilling its purpose and, in doing so, served as a model for all the tests that followed it. Hugo Munsterberg, a pioneer of industrial/organizational psychology [7], used, and advocated the use of, intelligence tests for personnel selection [8,9,10]. Historically, intelligence testing comprised a major branch of applied psychology due to it being widely practiced in schools, the workplace and the military [11,12,13,14], as it is today [15,16,17,18].

For as long as psychometric tests have been used to chart the basic structure of intelligence and predict criteria outside the laboratory (e.g., grades, job performance), there has been tension between emphasizing general and specific abilities [19,20,21]. Insofar as the basic structure of individual differences in cognitive abilities, these tensions have largely been resolved by integrating specific and general abilities into hierarchical models. In the applied realm, however, debate remains.

This state of affairs may seem surprising, as from the 1980s to the early 2000s, research findings consistently demonstrated that specific abilities were relatively useless for predicting important real-world outcomes (e.g., grades, job performance) once *g* was accounted for [22]. This point of view is perhaps best characterized by the moniker “Not Much More Than *g*” (NMM*g*) [23,24,25,26]. Nonetheless, even during the high-water mark of this point of view, there were occasional dissenters who explicitly questioned it [27,28,29] or conducted research demonstrating that sometimes specific abilities *did* account for useful incremental validity beyond *g* [30,31,32,33]. Furthermore, when surveys explicitly asked about the relative value of general and specific abilities for applied prediction, substantial disagreement was revealed [34,35]. Since the apogee of NMM*g*, there has been a growing revival of using specific abilities to predict applied criteria (e.g., [20,36,37,38,39,40,41,42,43,44,45,46,47,48,49]). Recently, there have been calls to investigate the applied potential of specific abilities (e.g., [50,51,52,53,54,55,56,57]), and personnel selection researchers are actively reexamining whether specific abilities have value beyond *g* for predicting performance [58]. The research literature supporting NMM*g* cannot be denied, however, and the point of view it represents retains its allure for interpreting many practical findings (e.g., [59,60]). The purpose of this special issue is to continue the “great debate” about the relative practical value of measures of specific and general abilities.

We solicited two types of contributions for the special issue. The first type of invitation was for nonempirical theoretical, critical or integrative perspectives on the issue of general versus specific abilities for predicting real-world outcomes. The second type was empirical and inspired by Bliese, Halverson and Schriesheim’s [61] approach: We provided a covariance matrix and the raw data for three intelligence measures from a Thurstonian test battery and school grades in a sample of German adolescents. Contributors were invited to analyze the data as they saw fit, with the overarching purpose of addressing three major questions:

- Do the data present evidence for the usefulness of specific abilities?
- How important are specific abilities relative to general abilities for predicting grades?
- To what degree could (or should) researchers use different prediction models for each of the different outcome criteria?

In asking contributors to analyze the same data according to their own theoretical and practical viewpoint(s), we hoped to draw out assumptions and perspectives that might otherwise remain implicit.

## 2. Data Provided

We provided a covariance matrix of the relationships between scores on three intelligence tests from a Thurstonian test battery and school grades in a sample of 219 German adolescents and young adults who were enrolled in a German middle, high or vocational school. The data were gathered directly at the schools or at a local fair for young adults interested in vocational education. A portion of these data were the basis for analyses published in Lang and Lang [62].

The intelligence tests came from the Wilde Intelligence test—a test rooted in Thurstone’s work in the 1940s that was developed in Germany in the 1950s with the original purpose of selecting civil service employees; the test is widely used in Europe due to its long history, and is now available in a revised version. The most recent iteration of this battery [63] includes a recommendation for a short form that consists of the three tests that generated the scores included in our data. The first test (“unfolding”) measures figural reasoning, the second consists of a relatively complex number-series task (and thus also measures reasoning), and third comprises verbal analogies. All three tests are speeded, meaning missingness is somewhat related to performance on the tests.

Grades in Germany are commonly rated on a scale ranging from very good (6) to poor (1). Poor is rarely used in the system and sometimes combined with insufficient (2), and thus rarely appears in the data supplied. The scale is roughly equivalent to the American grading system of A to F. The data include participants’ sex, age, and grades in Math, German, English and Sports.

We originally provided the data as a covariance matrix and aggregated raw data file but also shared item data with interested authors. We view them as fairly typical of intelligence data gathered in school and other applied settings. 

## 3. Theoretical Motivation

We judged it particularly important to draw out contributors’ theoretical and practical assumptions because different conceptualizations of intelligence require different approaches to data analysis in order to appropriately model the relations between abilities and criteria. Alternatives to models of intelligence rooted in Spearman’s original theory have existed almost since the inception of that theory (e.g., [64,65,66,67,68]), but have arisen with seemingly increasing regularity in the last 15 years (e.g., [69,70,71,72,73,74]). Unlike some other alternatives (e.g., [75,76,77,78,79]), most of these models do not cast doubt on the very existence of a general psychometric factor, but they do differ in its interpretation. These theories intrinsically offer differing outlooks on how *g* relates to specific abilities and, by extension, how to model relationships among *g*, specific abilities and practical outcomes. We illustrate this point by briefly outlining how the two hierarchical factor-analytic models most widely used for studying abilities at different strata [73] demand different analytic strategies to appropriately examine how those abilities relate to external criteria. 

The first type of hierarchical conceptualization is the higher-order (HO) model. In this family of models, the pervasive positive intercorrelations among scores on tests of specific abilities are taken to imply a “higher-order” latent trait that accounts for them. Although HO models (e.g., [80,81]) differ in the number and composition of their ability strata, they ultimately posit a general factor that sits atop their hierarchies. Thus, although HO models acknowledge the existence of specific abilities, they also treat *g* as a construct that accounts for much of the variance in those abilities and, by extension, whatever outcomes those narrower abilities are predictive of. By virtue of the fact that *g* resides at the apex of the specific ability hierarchies in these models, those abilities are ultimately “subordinate” to it [82].

A second family of hierarchical models consists of the bifactor or nested-factor (NF) models [30]. Typically, in this class of models a general latent factor associated with all observed variables is specified, along with narrower latent factors associated with only a subset of observed variables (see Reise [83] for more details). In the context of cognitive abilities assessment, this general latent factor is usually treated as representing *g*, and the narrower factors interpreted as representing specific abilities, depending upon the content of the test battery and the data analytic procedures implemented (e.g., [84]). As a consequence, *g* and specific ability factors are treated as uncorrelated in NF models. Unlike in HO models, these factors are not conceptualized as existing at different “levels”, but instead are treated as differing along a continuum of generality. In the NF family of models, the defining characteristic of the abilities is breadth, rather than subordination [82]. 

**Figure 1:**
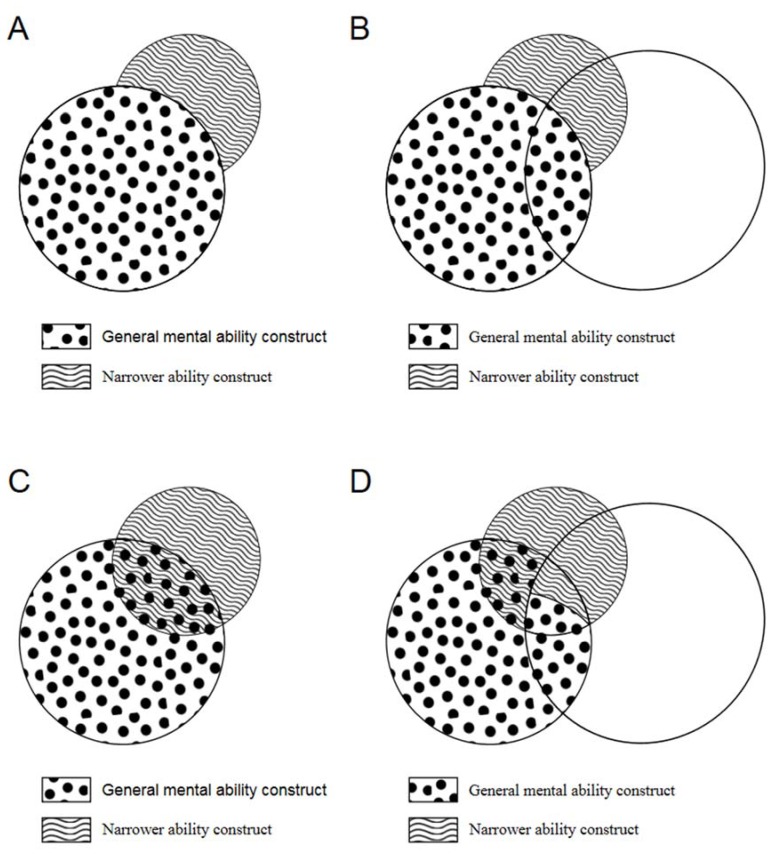
This figure depicts a simplified scenario with a single general mental ability (GMA) measure and a single narrow cognitive ability measure. As shown in Panel A, higher-order models attribute all shared variance between the GMA measure and the narrower cognitive ability measure to GMA. Panel B depicts the consequence of this type of conceptualization: Criterion variance in job performance jointly explained by the GMA measure and the narrower cognitive ability measure is solely attributed to GMA. Nested-factors models, in contrast, do not assume that the variance shared by the GMA measure and narrower cognitive ability measure is wholly attributable to GMA and distributes the variance across the two constructs (Panel C). Accordingly, as illustrated in Panel D, criterion variance in job performance jointly explained by the GMA measure and the narrower cognitive ability measure may be attributable to either the GMA construct or the narrower cognitive ability construct. Adapted from Lang et al. [20] (p. 599).

Lang et al. [20] illustrated that whether an HO or NF model is chosen to conceptualize individual differences in intelligence has important implications for analyzing the proportional relevance of general and specific abilities for predicting outcomes. When an HO model is selected, variance that is shared among *g*, specific abilities and a criterion will be attributed to *g*, as *g* is treated as a latent construct that accounts for variance in those specific abilities. As a consequence, only variance that is not shared between *g* and specific abilities is treated as a unique predictor of the criterion. This state of affairs is depicted in terms of predicting job performance with *g* and a single specific ability in panels A and B of Figure 1. In these scenarios, a commonly adopted approach is hierarchical regression, with *g* scores entered in the first step and specific ability scores in the second. In these situations, specific abilities typically account for a small amount of variance in the criterion beyond *g* [19,20].

When an NF model is selected to conceptualize individual differences in intelligence, *g* and specific abilities are treated as uncorrelated, necessitating a different analytic strategy than the traditional incremental validity approach when predicting practical criteria. Depending on the composition of the test(s) being used, some data analytic approaches include explicitly using a bifactor method to estimate *g* and specific abilities, and predicting criteria using the resultant latent variables [33], extracting *g* from test scores first and then using the residuals representing specific abilities to predict criteria [37], or using relative-importance analyses to ensure that variance shared among *g*, specific abilities and the criterion is not automatically attributed to *g* [20,44,47]. This final strategy is depicted in panels C and D of Figure 1. When an NF perspective is adopted, and the analyses are properly aligned with it, results often show that specific abilities can account for substantial variance in criteria beyond *g* and are sometimes even more important predictors than *g* [19].

The HO and NF conceptualizations are in many ways only a starting point for thinking about how to model relations among abilities of differing generality and practical criteria. Other approaches in (or related to) the factor-analytic tradition that can be used to explore these associations include the hierarchies of factor solutions method [73,85], behavior domain theory [86], and formative measurement models [87]. Other treatments of intelligence that reside outside the factor analytic tradition (e.g., [88,89]) and treat *g* as an emergent phenomenon represent new challenges (and opportunities) for studying the relative importance of different strata of abilities for predicting practical outcomes. The existence of these many possibilities for modeling differences in human cognitive abilities underscores the need for researchers and practitioners to select their analytic techniques carefully, in order to ensure those techniques are properly aligned with the model of intelligence being invoked. 

## 4. Editorial Note on the Contributions

The articles in this special issue were solicited from scholars who have demonstrated expertise in the investigation of not only human intelligence but also cognitive abilities of differing breadth and their associations with applied criteria. Consequently, we believe this collection of papers both provides an excellent overview of the ongoing debate about the relative practical importance of general and specific abilities, and substantially advances this debate. As editors, we have reviewed these contributions through multiple iterations of revision, and in all cases the authors were highly responsive to our feedback. We are proud to be the editors of a special issue that consists of such outstanding contributions to the field.
